# New KCNN4 Variants Associated With Anemia: Stomatocytosis Without Erythrocyte Dehydration

**DOI:** 10.3389/fphys.2022.918620

**Published:** 2022-08-08

**Authors:** B. Allegrini, S. Jedele, L. David Nguyen, M. Mignotet, R. Rapetti-Mauss, C. Etchebest, O. Fenneteau, A. Loubat, A. Boutet, C. Thomas, J. Durin, A. Petit, C. Badens, L. Garçon, L. Da Costa, H. Guizouarn

**Affiliations:** ^1^ Université Côte d’Azur, CNRS, INSERM, iBV, Nice, France; ^2^ Université Paris Cité and Université des Antilles, Inserm, BIGR, Paris, France; ^3^ Université Paris Cité, Paris, France; ^4^ AP-HP, Service d’Hématologie Biologique, Hôpital R. Debré, Paris, France; ^5^ Hôpital Saint Nazaire, Saint-Nazaire, France; ^6^ CHU Nantes, Service Oncologie-hématologie et Immunologie Pédiatrique, Nantes, France; ^7^ Sorbonne Université, AP-HP, Hôpital Armand Trousseau, Service d'Hématologie Oncologie Pédiatrique, Paris, France; ^8^ Aix Marseille Univ, INSERM, MMG, Marseille, France; ^9^ AP-HM, Department of Genetic, Marseille, France; ^10^ Université Picardie Jules Verne, Unité EA4666 Hematim, Amiens, France; ^11^ CHU Amiens, Service d'Hématologie Biologique, Amiens, France

**Keywords:** Hereditary Xerocytosis, Stomatocytosis, red blood cell, Gardos, KCNN4

## Abstract

The K^+^ channel activated by the Ca^2+^, KCNN4, has been shown to contribute to red blood cell dehydration in the rare hereditary hemolytic anemia, the dehydrated hereditary stomatocytosis. We report two *de novo* mutations on *KCNN4*, We reported two *de novo* mutations on *KCNN4*, V222L and H340N, characterized at the molecular, cellular and clinical levels. Whereas both mutations were shown to increase the calcium sensitivity of the K^+^ channel, leading to channel opening for lower calcium concentrations compared to WT KCNN4 channel, there was no obvious red blood cell dehydration in patients carrying one or the other mutation. The clinical phenotype was greatly different between carriers of the mutated gene ranging from severe anemia for one patient to a single episode of anemia for the other patient or no documented sign of anemia for the parents who also carried the mutation. These data compared to already published KCNN4 mutations question the role of KCNN4 gain-of-function mutations in hydration status and viability of red blood cells in bloodstream.

## Introduction

The rare disease dehydrated hereditary stomatocytosis (DHSt or hereditary xerocytosis, HX) is an autosomal dominant hemolytic anemia, characterized by an alteration of the cation permeability of red blood cell (RBC) (Badens and Guizouarn, 2016; Gallagher, 2017; Flatt and Bruce, 2018). In 2012, the identification of *PIEZO1* mutations in families suffering DHSt paved the way toward the genetic cause of this long-known hemolytic anemia (Zarychanski et al., 2012; Albuisson et al., 2013; Andolfo et al., 2013). Three years later, with the identification of mutations in *KCNN4*, another genetic cause of DHSt was claimed: “two genetic causes for a single RBC pathology” (Grygorczyk and Mohandas, 2015). KCNN4 is a Ca^2+^-activated K^+^ channel (also named SK4 or Gardos channel in RBC) (Gárdos et al., 1977; Maher and Kuchel, 2003) and PIEZO1 is a non-selective cation channel, permeable to Na^+^ and K^+^ and divalent cations Ca^2+^ and Mg^2+^ (Gnanasambandam et al., 2015) activated by mechanical stimuli (Coste et al., 2012). Numerous gain-of-function mutations in *PIEZO1* were identified in families with DHSt (Picard et al., 2019; More et al., 2020) and some of them were shown to modify the channel gating leading to longer open state that should increase Ca^2+^ concentration in RBC (Albuisson et al., 2013; Cahalan et al., 2015; Danielczok et al., 2017; Glogowska et al., 2017). This Ca^2+^ concentration rise can activate KCNN4, which leads to RBC dehydration (Syeda et al., 2015). The large anion conductance of erythrocyte membrane allows the loss of K^+^ when intracellular Ca^2+^ increase opens KCNN4. This KCl loss is accompanied by osmotically linked water. The Ca^2+^-induced K^+^ loss, named the Gardos effect, is of primary importance in RBC physiology (Gárdos et al., 1977; Maher and Kuchel, 2003). These cells do not regulate their volume. Hence, the dehydration resulting from KCNN4 activation is irreversible and this is expected to alter the RBC rheological properties. In sickle cell disease, the Gardos effect contributes to RBC sickling, the S-S hemoglobin having a greater tendency to polymerize following dehydration than A-A hemoglobin (Brugnara et al., 1993; De Franceschi et al., 1994). This mechanism has also been proposed to participate in the senescence of RBC. In a recent study, Rogers and Lew suggested that successive micro-activations of KCNN4 slowly dehydrate the RBC contributing to its densification, a marker of senescence (Rogers and Lew, 2021). In the case of PIEZO1 gain-of-function mutations linked to DHSt, a chronic stimulation of KCNN4 is considered as the cause of the observed RBC dehydration. In patients’ RBC with different PIEZO1 mutations, KCNN4 activation appears as the sole effector of this dehydration (Rapetti-Mauss et al., 2017).

In patients’ RBC carrying KCNN4 mutations, the channel conductance was increased (Rapetti-Mauss et al., 2016; Rivera et al., 2017; Fermo et al., 2020). Moreover, the mutations changed channel gating mainly by modifying its Ca^2+^ sensitivity (Garneau et al., 2009; Rapetti-Mauss et al., 2016).

Despite leading to a more active channel, the gain-of-function mutations in *KCNN4* are not systematically linked to RBC dehydration, and routine hematological tests failed to clearly diagnose DHSt (Picard et al., 2021). Nonetheless, these *KCNN4* mutations are associated with anemia that is often severe, especially in childhood and fetal life (Rapetti-Mauss et al., 2015).

Our present study was designed to reinforce our knowledge about KCNN4 mutations and DHSt. Two *de novo* KCNN4 mutations were identified in two unrelated families leading to the amino acid substitution V222L and H340N. This later had already been reported in a genetic screen but had not been functionally characterized (Andolfo et al., 2021). The two mutations were characterized by expression in HEK293T cells and it was observed that they changed the channel gating by calcium. Confirming previous observation with the majority of the KCNN4 mutations, our data did not correlate KCNN4 gain-of-function mutations with RBC dehydration, raising the question of classifying this pathology as a DHSt. Moreover, it emphasized the difficulty to diagnose altered RBC permeability facing KCNN4 mutations and the great variability in RBC phenotypes associated with KCNN4 gain-of-function mutations.

## Materials and Methods

**Patients:** Patients had been referred to our Hematology Diagnostic laboratory (AP-HP) for phenotypic and genotypic explorations. The lab is quality certified for molecular screening analysis for “targeted-NGS red cell and erythropoiesis defects” (Cofrac Iso15189) and labelized “Reference Medical Biological Laboratory” for red cell membrane diagnosis (LBMR July 2021). The patients exhibited chronic hemolytic anemia/hemolysis signs or had been followed up for uncharacterized red blood cell membrane disorder.

**Red cell and reticulocyte indices, EMA test, and Ektacytometry:** All blood samples were collected on EDTA and shipped at 4°C after blood was drawn along with a blood smear. Samples should be delivered to our laboratory within 48h after blood collection. RBC indices including hemoglobin concentration, hematocrit, mean cell volume (MCV), mean corpuscular hemoglobin concentration (MCHC), mean hemoglobin content (MHC), RBC volume distribution (RDW), and reticulocyte count and distribution have been measured for each sample using a hematological analyzer (XN, Sysmex, Kobe, Japan). Blood smears stained with May Grümwald Giemsa (MGG) were carefully examined and blind diagnostics of RBC morphology abnormalities were validated independently by two cytologists prior to additional analysis. The EMA test has been performed according to the recommendation (Girodon et al., 2008; Da Costa et al., 2016) with modification (Da Costa et al., 2016). The mean fluorescence intensity (MFI) for each sample was compared to three age-matched controls collected on the same day. A ratio of the mean fluorescence for patient RBC to the mean fluorescence for the three controls was derived (mean of three age-matched control MFI–patient MFI/mean of three age-matched control MFI). Ektacytometry LoRRca MaxSis (Mechatronics instruments BV^®^, Zwaag, Netherland) has been performed as previously described (Mohandas and Chasis, 1993; Mohandas and Gallagher, 2008; Da Costa et al., 2016). Blood samples (minimum of 100 μl) were analyzed by ektacytometry freshly and in any case before 48h after blood sample collection. Briefly, samples were subjected to increasing shear stress and an osmotic gradient and the laser diffraction pattern through the RBC suspension were recorded. The RBC shape goes from circular to elliptical as shear stress increases. From these measurements, a deformability or elongation index for the cells can be derived. Three distinct features of the osmotic gradient ektacytometry profiles are the Omin, the DImax, and the O’ or hyperpoints. The Omin point corresponds to the osmolarity at the minimal deformability in hypoosmolar area or at the osmolarity when 50% of the RBC hemolyzed during the regular osmotic resistance test. It reflects the surface-to-area ratio. DImax corresponds to the maximal deformability index or elongation index (EI). The hyperpoint or O’ corresponds to the osmolarity at half of the DImax and reflects the hydration state of the cells. Ektacytometry enables simultaneous analysis of three major RBC properties, RBC cell geometry, viscosity, and deformability, under the osmoscan application of the ektacytometer.

**Genotype characterization:** Genomic DNA was extracted from blood lymphocytes. Written informed consent was obtained from affected individuals and/or parents prior to inclusion in this study, which was performed in accordance with the ethical standards of the Declaration of Helsinki. The targeted New Generation Sequencing (t-NGS), developed in the lab, is a Roche “NimbleGen SeqCap EZ” library and an illumina flowcell (Flowcell standard 2*150) with a library of 187 genes including 93 genes for red cell disorders and erythropoiesis defects. Among them 21 genes were for red cell membrane disorders. The sequences have been run on a Miseq or a Nextseq in a genetic platform (Pr A. Verloes, Genetic Department, R. Debré hospital, Paris). Sequences have been analyzed on CLC Biomedical Work Bench and allelic variations have been interpreted with Qiagen Clinical Insight (QCI), Alamut visual (Sophia Genetics), and Varsome (Kopanos et al., 2019; Baux et al., 2021).

**Red cell cation content and volume measurements**: Fresh venous blood was obtained by venipuncture in EDTA collecting tubes from informed patients and healthy volunteers. Blood samples were received in the laboratory within 24–48 h traveling at room temperature. For vanadate experiments: blood was washed four times at room temperature in a medium containing (in mM): NaCl (145), KCl (5), MgSO_4_ (2), CaCl_2_ (1), and Hepes/NaOH, pH 7.4 (10). Red cell suspension was then incubated at room-temperature 25% hematocrit with 0.5 mM ouabain, and 5 mM vanadate was added alone or with 4 µM Senicapoc. A few minutes before sampling time, 400 µl of cell suspension was taken to fill three nylon tubes that were centrifuged for 10 min at 4°C, 20 000 g at the exact sampling time. The pellet of red cells was extracted and immediately weighted. Then, dry weight was measured after overnight heating (80°C). Water content was calculated with a correction of 3.64% corresponding to the trapped medium between packed cells. Intracellular ions were extracted from dried pellets by overnight incubation at 4°C in 5 ml milliRho water (Millipore). Two percent perchloric acid was then added to precipitate proteins, samples were centrifuged for 10 min 20 000 g, and supernatant was collected for Na^+^ and K^+^ quantification by flame spectroscopy with an Eppendorf ELEX6361.

**Intracellular Ca** ^**2+**^ **measurement**: RBCs were washed two times (800 g, 5 min, and 4°C) in Ringer without Ca^2+^ to remove buffy coat by aspiration. Four microliters of packed RBC were loaded with 2.5 µl of 1 mM Fluo-4 AM stock solution in 500 µl Ringer without Ca^2+^, 37°C, and 30 min. The Fluo4-loaded RBC suspension was directly used to quantify intracellular Ca^2+^ concentration (25 µl of RBC suspension in 975 µl Ringer without Ca^2+^ in FACS tubes) by measuring fluorescence with a FACS Fortessa BD. Internal RBC fluorescence was assessed on RBC treated without Fluo-4 AM. For vanadate experiments, the Fluo4-loaded RBC suspension was diluted 40 times in Ringer with 1 mM Ca^2+^ and 5 mM vanadate at time 0 and intracellular fluorescence was measured with FACS Fortessa BD as a function of time.

**HEK293T cells transfection**: HEK293T cells were grown in DMEM glutamax (Gibco) 10% FBS penicillin–streptomycin. Cells were co-transfected with WT or point-mutated pcDNA3-KCNN4-HA and pIRES-eGFP (ratio 10:1) using CaPO_4_. WT pcDNA3-KCNN4-HA was a kind gift of Len Kaczmarek laboratory. Sixteen hours later, cells were washed twice with PBS, and patch clamp was done on fluorescently labeled cells. Point mutations were done by PCR on pcDNA3-KCNN4-HA with the proofreading DNA polymerase pfu-Turbo and primers covering 16 nucleotides upstream and downstream the single point mutation C1018 A for H340N or G649C for V222L. The pcDNA3-KCNN4-HA H340N or V222L clones used in the study were sequenced entirely.

**Protein expression assay**: HEK293T cells were grown to 70% confluence in DMEM glutamax 10% FBS penicillin–streptomycin in 60 mm Petri dishes (Starsted) and transfected with CaPO4 with 5 µg of DNA: pcDNA3-KCNN4-HA WT, V222L, or H340N per 5 ml cell culture. Transfected medium was removed after 6h. After 24h of expression, cells were biotinylated following manufacturer instructions (Pierce cell surface protein assay), and then lysed. The lysate was loaded on avidin-agarose beads (Pierce cell surface protein assay) to isolate the biotinylated fraction. Total fraction and biotinylated fraction were subjected to SDS-PAGE western blot. Migration of proteins was made at 120 V during 90 min in 10% acrylamide gel. Proteins were then transferred to PVDF using wet transfer protocol for 1h at 100 V and blocked in blocking solution (BS: 5% low-fat milk in TBS-tween 0.1%). Immunolabelling was done using primary antibodies: anti-KCNN4 (Proteintech, Rabbit, 1:1,000), anti-Ecadherin (Cell Signaling, Mouse, 1:5,000), and anti-GAPDH (Calbiochem, Mouse, 1:200,000) for 1 h 30 min at room temperature in BS and HRP coupled secondary antibodies for 50 min at RT using: anti-rabbit (1:2,000, DAKO) and anti-mouse (1:5,000, DAKO). HRP-labeled proteins were revealed with Enhanced Chemiluminescent solution (Millipore) with a Fusion FX EDGE.

**Patch-clamp electrophysiology**: Glass pipettes (Brand, Wertheim, Germany) with final resistance of 3–5 MΩ were made on a horizontal pipette puller (P-97, Sutter Instrument, Navato, CA). All patch-clamp experiments were performed with a PC-controlled EPC9 patch-clamp amplifier (HEKA, Lambrecht/Pfalz, Germany). Currents were acquired and analyzed with Pulse and Pulsefit software (HEKA).

Currents were measured in whole-cell configuration with bath solution (mM): NaCl (145), CaCl_2_ (2), KCl (5), MgCl_2_ (1), and HEPES (10), pH 7.4 adjusted with NaOH. Pipette solution (mM): KCl (145), MgCl_2_ (1), HEPES (1), and EGTA (1), pH 7.2 adjusted with KOH. Free Ca^2+^ concentrations were adjusted by adding CaCl_2_ using Ca-EGTA Calculator v1.3. Currents were measured at room temperature using a ramp protocol from -120 to +80 mV from a holding potential of -60 mV (sampling frequency 10 kHz; filtered 5 kHz).

**KCNN4/CaM complex modeling:** Three full-length models of the KCNN4/Calmodulin (CaM) complex were constructed by assembling KCNN4 C-ter helices (residues 376 to 415, PDB ID: 6D42) (Ji et al., 2018) to the three cryo-EM structures catching the main part of the channel in three distinct conformational states (inactivated, activated/closed, and activated/open) with CaM and Ca^2+^ ions (PDB IDs: 6CNM, 6CNN, and 6CNO, respectively) (Lee and MacKinnon, 2018). Missing CaM N-lobe in the first structure was modeled by adding the N-lobe of the second structure and changing some torsion angles in CaM linker region, so that the CaM N-lobe is kept in solvent as described in the associated publication. Missing loops and residues were modeled using MODELLER (Webb and Sali, 2016) and residue protonation states were predicted at pH 7 using PROPKA (Olsson et al., 2011; Søndergaard et al., 2011). Models of V222L and H340N KCNN4 mutants were obtained by replacing the side-chain of the mutated residue. Resulting structures were embedded in pure symmetric POPC membranes with 150 mM of KCl using CHARMM-GUI Membrane Builder server (Wu et al., 2014) and further relaxed using 1 µs molecular dynamics simulations carried out with GROMACS (Abraham et al., 2015). Models were represented using PyMol (Shrödinger L., DeLano W.2020).

**Residue conservation**: Residue conservation analysis was based on the comparison of 3944 sequences collected from the NCBI non-redundant protein sequence database using blastp tool with human KCNN4 sequence (UniProt ID: O15554) as input query. Only sequences with sizes lower than 900 residues or with query coverage values higher than 66% were kept. Synthetic constructs were removed. Multiple alignments and associated weblogos were obtained using the EBI clustal omega tool (Sievers et al., 2011) and Weblogo server (Crooks et al., 2004). Conservation frequency of the KCNN4 residues was calculated as the occurrence ratio of the KCNN4 residue at the given position in the multiple sequence alignment.

## Results

### Clinical Description of the Cases

Two subjects from two unrelated families were enrolled in this study after informed consent. The proband A had history of severe anemia requiring transfusion every 5–6 weeks since the age of 2 months when anemia was diagnosed. There was no splenomegaly, iron chelation was started at the age of 3 years. Blood withdrawal for permeability measurements was done on two occasions 3 months after the last transfusion.

The proband B was born after an uneventful pregnancy from non-consanguineous parents and delivery at 40 weeks by caesarean operation. At 9 months in a routine examination, he presented icterus and pallor requiring hematologic investigation in hospital. Results are presented in Table 1. Hemoglobin electrophoresis was normal (HbF 3.3%), G6PD and PK were normal, with no sign of infection, and Coombs test was negative. Bone marrow examination showed erythroid hyperplasia with discrete dyserythropoiesis. He was transfused and a steroid therapy had been started for 3 months. At the end of the steroid therapy, hemoglobin was stabilized. Growth and psychomotor development were normal and 4 years later, hematological parameters were normal (Table 1).

**TABLE 1:** Hematologic data collected from publications describing patients with V282M or R352H KCNN4 mutation (^1^ Andolfo et al. 2015,^2^ Waldstein et al. 2021,^3^ Rapetti-Mauss et al. 2015) and from the new patients with V222L or H340N KCNN4 mutations.

Parameters	Normal range	V282M Worcester^1^		R352H Naples^1^			R352H Milan^1^		R352H American^2^			R352H Marseille^3^		R352H Paris^3^		V222L Nantes		H340N Paris		
		III.5	IV.5	F		Child	Father	Child (F)	Mother	Daughter	Son	Mother	Child (M)	Mother	Child (M)	Proband A (F)		Proband B (M)		
				Pre-splen	Post-splen															
Age (years)	—	56	21	13	40	10	40	1	61	39	34		0.4	25	2	3.5	4.2	0.75	1.3	4.3
Hb (g/dl)	11.5–16.5	13.3	12.5	10.4	9	10.5	10.5	9.9	10.5	11.1	9.3	8.5	9.8	11	10.4	7.5	8.9	7.7	12.3	13.7
RBC (10^12^/L)	3.8–5.8	3.9	3.3	3	2.5	3.6										2.72	3.11	2.4	4.23	
Hct (%)	37–47	37	35.6	30.5	28.5	31.3										22.5	25.4	21.6		
MCV (fL)	76–96	103	96	101	112	86	108	81.5	106.5	99.9	98.8	109	87.9	93.1	86.9	82.8	81.7	90	77.3	79.6
MCH (pg/cell)	27–32	–	33.7	34.3	35.4	28.8	35.8	29.1								27.6	28.6	32	29.1	
MCHC (g/dl)	30–35	–	36.1	34	31.6	33.5	33	35.7	34.1	33.9	32.9	35.4	35.6	36.1	36.5	33.3	35	35.6	37.6	
RDW (%)	11.5–15.5	–	14.7	–	14.5	4.1			15	16.1	21.2					14.8	14.1	—	10.1	
Retics %	0.5–2	6.5	12.2	11.8	17.7	6.3			12.6	6.7	>22					ND	ND	6.44	1.98	
Retic absolute count (10^3^/µl)	20–80	254	403	358	450	229						255	263	249	363	ND	ND	155		71.6

(F) refers to female and (M) to male.

### Genetics

**FIGURE 1:**
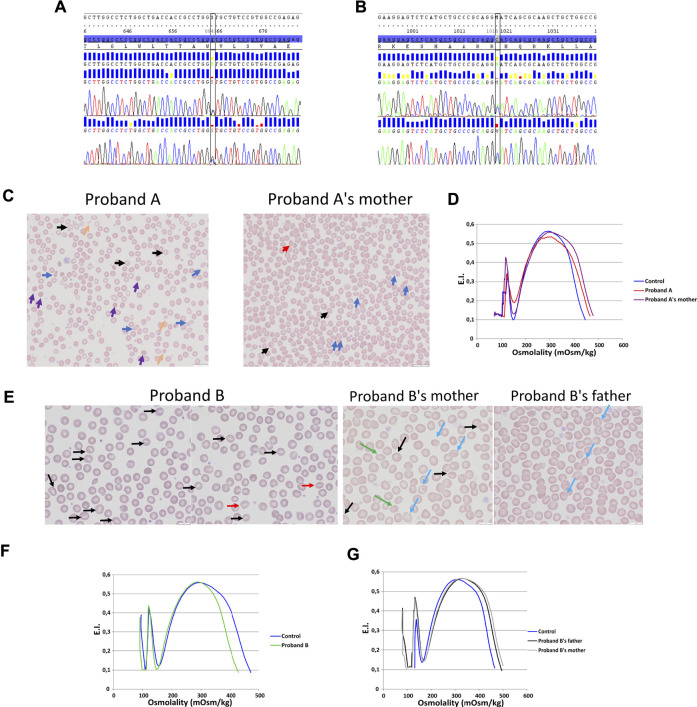
Blood smear, ektacytometry and genetics. DNA sequencing on proband A **(A)** and proband B **(B)**. **(C)**: Blood smear on proband A with her mother (mutation KCNN4 V222L). **(D)**: Ektacytometry on proband A with her mother and control. **(E)**: blood smear done on proband B RBC (mutation KCNN4 H340N) and his father and mother. Ektacytometry on proband B **(F)** and proband B’s mother's and father’s RBC **(G)**.

Two allelic variations have been identified in *KCNN4* gene (NM_002250.2) in two unrelated probands: 1) proband A carried a heterozygous missense allelic variation in exon 3 of *KCNN4*: c.664G > C; *p*.(Val222Leu) (Figure 1A), variant without significance [VUS or Class 3 (Richards et al., 2015; Amendola et al., 2016)], sift damaging, Mutation Taster disease causing, absent from the database. The variation has been inherited from the mother. 2) proband B carried a heterozygous missense allelic variation in exon 6 of *KCNN4*: c.1018C > A; *p*.(His340Asn) (rs76935412) (Figure 1B), likely pathogenic (class 4, Varsome *in silico* prediction) variant, sift damaging, Mutation Taster disease causing, level pathogenic, MetaLR damaging, allele frequencies of 0.16%. The variation has been inherited from the mother.

### Hematological Parameters

Table 1 compared hematological data coming from the new cases with H340N or V222L KCNN4 mutations with previously described RBC from patients carrying the R352H or V282M KCNN4 mutations. Anemia is a common feature of the different cases. This anemia is compensated in the family with V282M mutation showing a normal level of hemoglobin associated with hyper-reticulocytosis. The other cases exhibited a severe anemia not compensated by high reticulocytosis. The mutation H340N KCNN4 was correlated with a single episode of anemia which had not been further observed. In contrast, proband A with V222L KCNN4 mutation exhibited a severe uncompensated anemia associated with major dyserythropoiesis that could not be explained genetically, but a defect in iron/heme metabolism has been suggested.

MCHC is in the normal range or only few deciles above maximum. However, the MCHC has been reported at 37.6 g/dL in proband B at the time ektacytometry was performed (1.3 years) away from the hemolytic crisis (9 months). The size of RBC is most of the time larger than control (macrocytosis), while normal in probands A and B. These observations might result from the hyper-reticulocytosis: the maximum MCV is increased by 6–18% which could result from a 5–17% reticulocytes.

Blood smear showed anisopoikilocytosis (Figure 1C), with rare spherocytes (purple arrows), rare elliptocytes (blue arrows), rare stomatocytes (black arrows), few RBC fragments (yellow arrows), and erythroblastemia for proband A with V222L KCNN4 mutation. Proband A’s mother’s blood smear did not show noticeable erythrocyte anomaly, except rare elliptocytes and very rare stomatocytes (Figure 1C). The ektacytometry indicated altered RBC deformability for proband A without any shift in osmotic resistance (Figure 1D). Omin point was normal while hyperpoint was shifted to the right. Proband A’s mother’s RBC deformability was normal and hyperpoint exhibited the same shift to the right as her daughter. For proband B, blood smear strikingly exhibited few stomatocytes [black and red (smile feature) arrows] (Figure 1E). The ektacytometry indicated normal deformability with a shift to the left of the hyperpoint in accordance with the increased MCHC at 37.6 g/dL in proband B at the time of the study (Figure 1F). The mother and the father exhibited normal ektacytometry curves (Figure 1G) and the mother, who carried the same allelic variation as his son, exhibited only rare stomatocytes, rare target cells (blue arrows), and acanthocytes (green arrows), while the father exhibited only few target cells (blue arrows) (Figure 1E).

### Red Blood Cell Permeability

**FIGURE 2:**
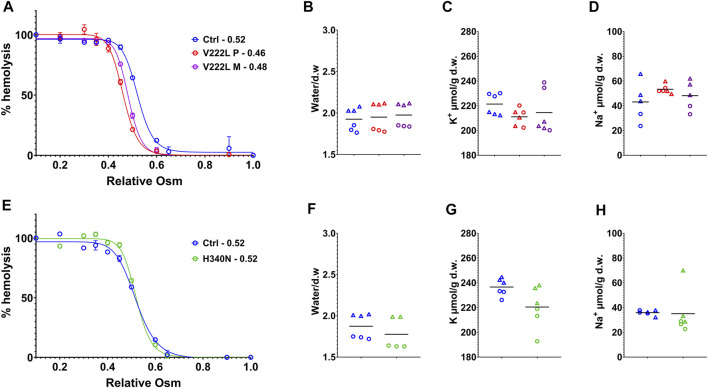
Control vs. mutated RBC analysis. RBC analysis was made on patients and control blood collected at the same time and traveled together to the laboratory. **(A,E)**: Osmotic resistance analysis made on total blood represented as percentage hemolysis function of relative NaCl concentration. Data are means ± SD illustrating one experiment done in triplicate on one blood shipment for each family. **(A)** Control in blue, V222L proband A in red and proband A’s mother in purple; **(E)** control in blue, H340N proband B in green. **(B,C,D,F,G,H)**: RBC were washed in Ringer (1 mM Ca^2+^), dried, and weighted for water content expressed in g of water/g of dry weight **(B,F)**, then solubilized for K^+^ **(C,G)** and Na^+^ **(D,H)** content measurements expressed in µmol/g of dry weight. Data showed the results coming from experiments performed on two blood shipments for each patient with corresponding controls (each experiment in triplicate, hence, six symbols of the same color per condition). Circles or triangles correspond to paired samples in the same shipment. From left to right along *x* axis in each plot: control (blue), proband A V222L (red), and proband A’s mother (purple) **(B,C,D)** and control (blue), proband B, and H340N (green) **(F,G,H)**. The bars represent the median. Statistical analyses were made with a Mann–Whitney test when comparing two conditions or Kruskal–Wallis if more than two. Neither of both gave statistical differences for any conditions.

RBC water, K^+^- and Na^+^-ion contents were measured following RBC washing in saline buffer containing calcium, 48h after blood collection in EDTA tubes. Figure 2 illustrates data coming from experiments corresponding to two different shipments of either proband A or proband B blood samples collected at different times. Osmotic resistance curves for proband A as for the mother with V222L mutation were slightly shifted to the left compared to control (Figure 2A). However, this shift is in the normal range and there was no change in water content between control and mutant (Figure 2B). The K^+^ and Na^+^ contents of RBC with V222L KCNN4 were not significantly different from control despite a tendency to increased Na^+^ content correlated with decreased K^+^ content in one blood sample (Figures 2C,D). Osmotic resistance curves showed no significant difference between control and H340N mutants (Figure 2E). This is correlated with no significant change in water content (Figure 2F). The K^+^ and Na^+^ contents were also similar between control and H340N mutants (Figures 2G,H). Hence, it was not possible to detect any significant change in water, K^+^, and Na^+^ permeability in RBC carrying KCNN4 mutations V222L or H340N compared to WT. Similar results were observed previously in RBC with R352H mutated KCNN4 (Andolfo et al., 2015; Glogowska et al., 2015; Rapetti-Mauss et al., 2015).

**FIGURE 3:**
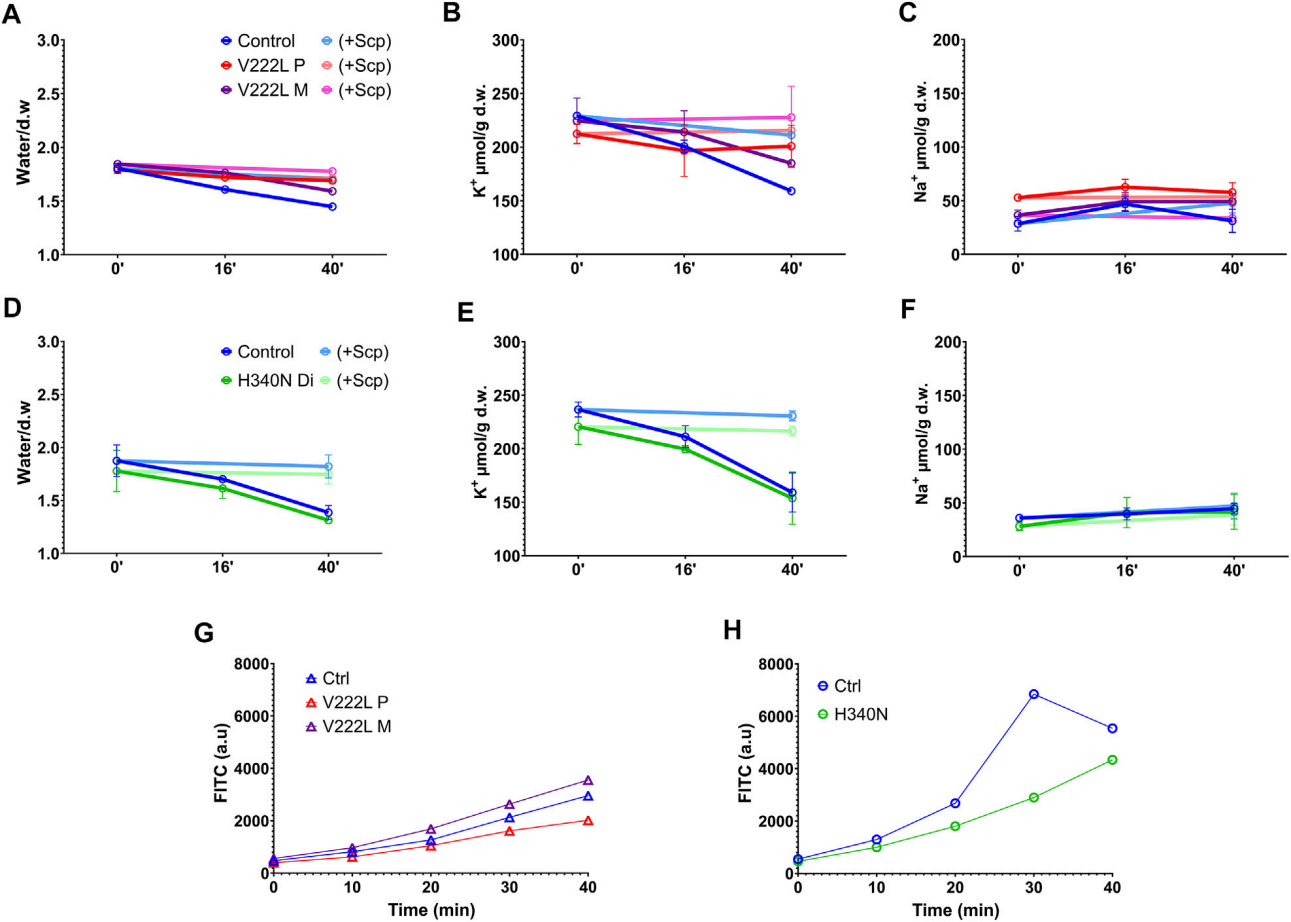
RBC ion, water, and Ca^2+^ contents in response to vanadate. Washed RBCs were submitted to 5 mM Vanadate with or without 4 µM Senicapoc treatment in the presence of 0.5 mM ouabain. Water, K^+,^ and Na^+^ contents were measured at different time points (0′, 16′ and 40′). Water content **(A,D)**, K^+^ content **(B,E)**, and Na^+^ content **(C,F)** are represented for control in blue, V222L proband A in red and proband A’s mother in purple: panel **(A,B,C)**, and control in blue, H340N proband B in green: panel **(D,E,F)**. Bright colors represented Senicapoc effect compared to untreated conditions. Data are given as mean ± SD of triplicate experiments done on either a single blood shipment for proband A [panel **(A,B,C)**] or two different blood shipments for proband B [panel **(D,E,F)**]. **(G,H)**: Fresh RBCs were washed in Ringer without calcium and incubated with 5 µM Fluo-4AM 30min at 37°C. Fluo-4AM-loaded RBCs were then incubated in Ringer solution with 1 mM Ca^2+^ and 5 mM vanadate. Fluorescence was measured with a FACS as a function of time. Data are given as FITC in arbitrary units, median of a single experiment, 10,000 events. The fluorescence linked to basal intracellular Ca^2+^ concentration was in arbitrary units, mean ± s.e.m.: 750 ± 9 for proband A, 1,167 ± 6 for proband A’s mother, and 839 ± 6 for control and 528 ± 5 for proband B compared to 533 ± 7 for control.

In order to unmask a possible difference in the activation of mutated KCNN4 compared to WT, control and mutant RBCs were challenged with vanadate, inhibitor of the Ca^2+^-ATPase pump. Blocking Ca^2+^ pump leads to intracellular Ca^2+^ increase that in turn activates KCNN4. This activation resulted in K^+^ loss that can be blocked by Senicapoc, inhibitor of KCNN4. A significant dehydration correlated with K^+^ loss was observed in control RBC 40 min after vanadate addition that was blocked by Senicapoc (Figures 3A,B D,E), confirming the involvement of KCNN4 in RBC water loss. The K^+^ drop was marked after 16 min with vanadate and negligible before 16 min. Vanadate did not change RBC Na^+^ content (Figures 3C,F). Following incubation with vanadate, the K^+^ content decreased in RBC with either V222L or H340N KCNN4 mutations, and this K^+^ loss was blocked by addition of Senicapoc. Following the same scheme as R352H KCNN4, a greater Ca^2+^ sensitivity of V222L and H340N mutants was expected to increase K^+^ loss within 16 min incubation with vanadate (Rapetti-Mauss et al., 2015). However, there was no difference between control and mutant RBC 16 min after vanadate addition. Strikingly, the K^+^ loss and dehydration were even reduced compared to control for proband A with V222L KCNN4 mutation (Figures 3B,C).

Intracellular Ca^2+^ rise following Ca^2+^ pump inhibition with vanadate depends on the Ca^2+^ leak allowing progressive Ca^2+^ uptake. Hence, the absence of differential activation of mutated KCNN4 compared to WT in RBC incubated with vanadate could be explained by variations in intracellular Ca^2+^ concentration between samples. Using the fluorescent probe Fluo4, intracellular Ca^2+^ concentration was measured in the different RBC incubated with vanadate (Figures 3G,H). Vanadate increased similarly intracellular calcium concentration in control and patients’ RBC in the different experiments.

### KCNN4 Expression in HEK293T Cells

**FIGURE 4:**
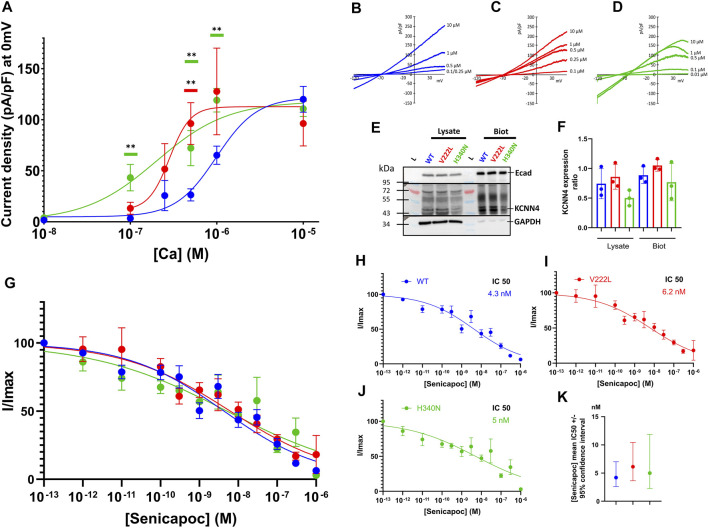
Calcium sensitivity of KCNN4 mutations V222L and H340N in HEK293T cells. **(A)** Current density (pA/pF) was measured in response to (Ca^2+^)_i_ for KCNN4 WT (blue), V222L (red), and H340N (green). Experimental values were fitted with a variable slope model (Hill equation) (Y = Y_min_ + (X^hillslope^)*(Y_max_- Y_min_)/(X^hillslope^ + EC50^Hillslope^). The resulting EC50 corresponds to the (Ca^2+^)_i_ at which half of the max current is reached. **(B–D)** Representative I/V curves for WT, V222L, and H340N at different Ca^2+^ concentrations (0.01–0.1–0.25–0.5–1, and 10 µM). Statistical analysis against WT at different Ca^2+^ concentrations was performed using a Kruskal–Wallis test followed by an uncorrected Dunn’s test, *n* = 7–12 (*: *p* < 0.05; **: *p* < 0.01). **(E)** Representative western blot showing total (Lysate) or only surface (Biot) protein expression of KCNN4 WT, V222L, and H340N in HEK293T-transfected cells. Total fraction corresponded to cell lysate and biotinylated fraction corresponded to membrane proteins. We used GAPDH as housekeeping for total fraction quantification (lysate) and E-cadherin for biotinylated fraction quantification. In KCNN4 labeling, the three bands between 43 kDa and 55 kDa corresponded to different glycosylated states of the protein. In lysate, the band at 72 kDa is non-specific. **(F)** Ratio KCNN4/GAPDH in lysate and KCNN4/ECadherin (biotinylation) were calculated from three independent Western Blots, taking the total KCNN4 signal. No significantly increased expression with V222L and H340N compared to WT KCNN4 was observed. Only H340N tended to have a reduced expression. Kruskal–Wallis test was used to determine statistical significance against WT. **(G)** Senicapoc sensitivity of KCNN4 WT or mutated. Normalized currents were measured in response to [Senicapoc] for WT (blue), V222L (red), and H340N (green). Experimental values were fitted with variable slope model (Hill equation): Y = 100/(1 + (IC50/X)^hillslope^). The resulting IC50 corresponded to 50% inhibition by Senicapoc; the mean value ±95% confidence interval of IC50 is represented in **(K)**, *n* = 15–21. For clarity purposes, fits are represented alone for each condition **(H,I,J)**. Statistical analyses were performed using a Kruskal–Wallis test followed by an uncorrected Dunn’s test, *n* = 15–21.

To study the effect of KCNN4 mutations on the channel activity, HEK293T cells were transfected with either WT KCNN4 or H340N KCNN4 or V222L KCNN4. It is observed that the maximal current intensity is not changed by the mutations (Figures 4A–D). However, the two mutants V222L and H340N were more sensitive to Ca^2+^ compared to WT with an IC50 = 0.28 and 0.20 µM, respectively (0.99 µM for WT). The expression at the plasma membrane of the different constructs was assessed by western blotting of biotinylated proteins. Immunodetection of E-cadherin was used as a reference for expression of endogenous membrane protein to compare the different conditions. All the constructs were similarly expressed at the plasma membrane (Figures 4E,F), ruling out an effect of the mutations on protein addressing the HEK293T plasma membrane. The mutants were sensitive to Senicapoc, blocker of KCNN4, showing a sensitivity similar to the WT (Figures 4G–K).

### KCNN4 Structure Analysis

**FIGURE 5:**
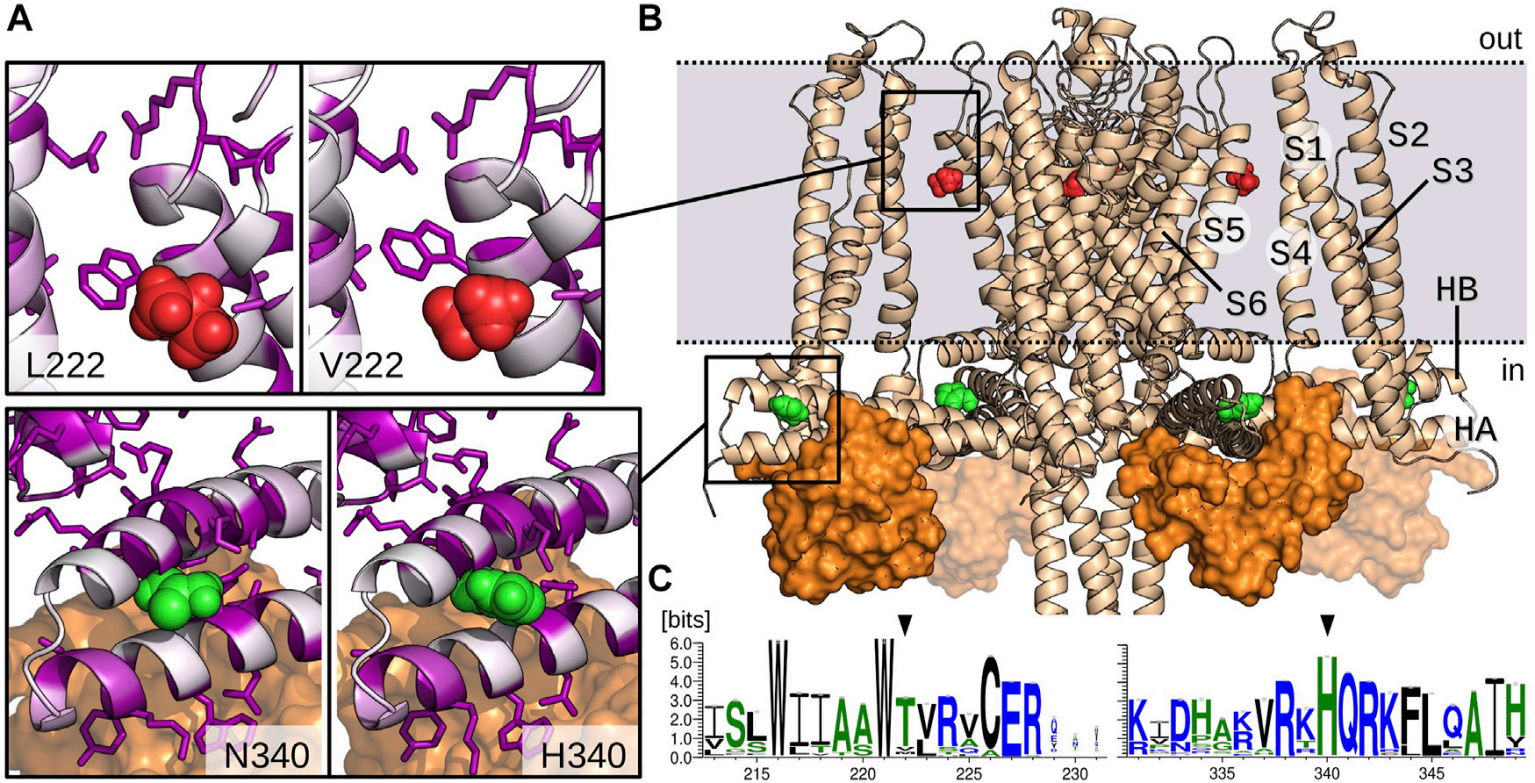
Model of KCNN4 showing V222 and H340 amino acids location. **(A)** Zoom on V222 (red) and H340 (green) environment in the activated/closed KCNN4/CaM model. Mutant models (L222 and N340) are presented in the left windows. KCNN4 residues are colored according to their conservation frequency (white to purple) in the multiple sequence alignment. Side chains of residues with a conservation percentage greater than 70% are represented. **(B)** Model of KCNN4/CaM complex based on cryo-EM structures. For clarity, only KCNN4 and C-lobes (orange surface) of the four CaM molecules are shown. Dashed lines delineate the position of plasma membrane upper and lower leaflets. **(C)** Weblogos of the KCNN4 sequence segments encompassing positions 222 and 340 (black arrows).

The two mutations are located in very different regions of the channel (Figures 5A,B). The multiple sequence analysis also showed drastic different profiles for V222L and H340N mutations in terms of conservation. Indeed, the weblogo established from this alignment (Figure 5C) designates H340 as a highly conserved residue (99%), in contrast the Valine in position 222 is poorly conserved (8%). This last position is actually mainly occupied by Threonine (83%). Leucine residue can be found but extremely rarely in the superfamily of KCNN4 (0.9%).

The 3D structure shows that H340 is located in the binding site with the constitutively bound calmodulin (CaM) C-lobe (Figure 5B), More precisely, H340 is carried by the intracellular HB helix, in the region facing the HA helix and close to the peripheral helices S2–S3 linker. H340 is in contact with residues E321, M324, F325, and H328 of HA and A336 and L343 of HB. V222 is located in the transmembrane part of the channel, in the middle of the interface bridging the S1/S4 peripheral helices with the S5/S6 helices that constitute the pore domain. V222 is in contact with both L154 and L157 situated in the peripheral S4 helix and with the conserved residue V266 from helix S6. It is also close to the interface between S1 and S5 helices involving highly conserved residues, i.e., V41 (87%) and E45 (93%) on one side and residues W221 (99%) and R228 (99%) on the other side.

Preliminary results obtained for the mutated modeled structures relaxed with molecular dynamics simulations did not show major changes in the environment of the residue, most local contacts being preserved. However, this does not preclude long range effects as observed through slight displacements of helices for the two mutants along the molecular dynamic simulations.

## Discussion

At the molecular level, it was shown that the KCNN4 mutations V222L and H340N altered the Ca^2+^ sensitivity of the K^+^ channel and could be classified as gain-of-function mutations. The IC50 is shifted toward lower intracellular Ca^2+^ concentrations suggesting that the mutated channels might be activated with a lower Ca^2+^ increase compared to WT channels. The functional changes observed for the two mutants may originate from two mechanisms: the first one involving V222L could be due to the fine regulation that exists between the peripheral helices and the pore helices during the transport; in contrast, the second one involving H340N may participate in the activation process itself, by changing the interplay between CaM and the channel. In this last case, it is important to note that other mutations (R352, A322, and S314) in CaM C-lobe-binding site have been reported.

Clinically, these two new KCNN4 mutations blurred a bit more the phenotype of KCNN4 gain-of-function mutations. From Table 1, it appears that KCNN4 gain-of-function mutations are associated with more or less compensated hemolytic anemia. For proband A with V222L mutation, the anemia seemed related to dyserythropoiesis and strikingly the mother carrying the same mutation had no history of anemia. For proband B with H340N mutation, the anemia was transient and again the mother carrying this mutation had never been followed for hematological disorder. The complete response observed under steroids, however, challenges the responsibility of the KCNN4 mutation alone in the pathophysiology of this transient anemia. This questions the role of these two KCNN4 gain-of-function mutations in proband A’s constitutive or proband B’s transient anemia. The hematologic disorder might result from the combination between the presence of KCNN4 gain-of-function mutations and a secondary trigger such as an infection, or a cumulative mutation on another gene involved in erythropoiesis, not included in the 93 “RBC genes” of the t-NGS library, or structural allelic variation not identified in t-NGS. A previous article reported the H340N KCNN4 mutation in a genetic study enrolling 155 patients with suspected hereditary anemia (Andolfo et al., 2021). This KCNN4 mutation was identified in two patients from different kindred and it was associated with either a SPTA1 mutation or two independent mutations on LARS2 and ABCB6. Unfortunately, no hematological or clinical data were given and we could not compare it with the present cases. Considering proband A (V222L mutation), the non-regenerative anemia may be due to either a non-identified intercurrent cause, or a role of KCNN4 during erythropoiesis, as described for PIEZO1-related DHSt (Caulier et al., 2020).

At the cell level, neither V222L nor H340N KCNN4 gain-of-function mutations did correlate with altered RBC hydration status: 48 h after withdrawal in EDTA collecting tubes, the osmotic resistance curves were within normal range. When assessed, the MCHC was normal or slightly above the maximal value, and the water, Na^+,^ and K^+^ contents were not significantly different from WT. Previous data with other KCNN4 mutations reported by us or others in different publications also failed to correlate KCNN4 mutations with RBC dehydration (Andolfo et al., 2015; Fermo et al., 2015; Rapetti-Mauss et al., 2015; Fermo et al., 2017; Waldstein et al., 2021).

The higher Ca^2+^ sensitivity of H340N and V222L KCNN4 as well as the other characterized mutants S314P and R352H (Rapetti-Mauss et al., 2015; Rivera et al., 2017; Fermo et al., 2020) suggested that these channels would be more often activated in RBC challenged with increasing Ca^2+^ concentration. This higher Ca^2+^ sensitivity of mutated KCNN4 was expected to induce RBC shrinking due to net KCl loss accompanied by water loss that is not compensated in human RBC which does not regulate their volume. It could be argued that to observe KCNN4 activity, Ca^2+^ has to be present in extracellular medium and its concentration should increase in the cell (Dyrda et al., 2010). In ektacytometry experiments as in osmotic resistance measurements, there was no Ca^2+^ in extracellular medium and the water uptake or the elongation resulted in no change or at worse a decrease in intracellular Ca^2+^ (due to water uptake). These experimental conditions do not promote KCNN4 activation. We have previously shown that osmotic resistance curves done on RBC carrying R352H KCNN4 mutation treated with the anticoagulant heparin, within hours following withdrawal, were shifted to the left compared to WT, indicating RBC dehydration (Rapetti-Mauss et al., 2015). A similar shift to the left on ektacytometry curves done on RBC with R352H or A322V KCNN4 was observed in another laboratory, indicating experimental conditions allowing KCNN4 activation in patient RBC compared to control WT (Mansour-Hendili et al., 2021). Moreover, at the time ektacytometry analysis was done on proband B, a shift to the left of the curve indicated dehydration that was corroborated by increased MCHC (Table 1 age 1.3 years). Hence, there might be a situation where the RBC carrying KCNN4 mutations would appear dehydrated. To unmask the consequences of KCNN4 gain-of-function mutations on RBC water homeostasis, Ca^2+^ must be present in extracellular medium and the RBC energy depleted: without ATP, the Ca^2+^ pump activity decreases and intracellular Ca^2+^ slowly increases leading to the activation of KCNN4. Gain-of-function KCNN4 mutations will result in a more rapid activation of the channel compared to WT. Thus, depending on 1) the energy status of RBC following blood withdrawal and 2) the presence of Ca^2+^ in medium, mutated KCNN4 could be activated and patient RBC dehydration could be observed. The fact that in EDTA collecting tubes within 24–48 h of withdrawal, patient RBC was within normal range for water content, suggested KCNN4 activity was kept silent in bloodstream. Alternatively, we can hypothesize that the RBC where KCNN4 was activated were dramatically dehydrated and immediately removed from circulation and could not be observed in blood samples. However, blood smear clearly indicated altered RBC shapes (Figure 1) that were even more visible when patients were splenectomized (Rapetti-Mauss et al., 2015). In addition, the ektacytometry indicated in some patients an increased RBC fragility (diminution of the maximum and increase in the minimum elongation index, proband A Figure 1). Nonetheless, these altered shapes or RBC fragility could not be linked to changes in RBC hydration status, or K^+^ and Na^+^ contents. This questions the relevance of connecting the hydration status and RBC fragility in case of KCNN4 gain-of-function mutations.

Moreover, it reinforces the phenotype distinction between KCNN4 gain-of-function mutations and gain-of-function mutations in PIEZO1. In similar experimental condition (EDTA collecting tubes), PIEZO1-mutated RBC is most of the time dehydrated and ektacytometry or osmotic resistance curves show a leftward shift of the curves compared to control (Rapetti-Mauss et al., 2017; Picard et al., 2019; More et al., 2020; Andolfo et al., 2021). The RBC dehydration linked to PIEZO1 gain-of-function mutations was explained by a more frequent stimulation of KCNN4 (Albuisson et al., 2013), leading to a new RBC volume homeostasis. The role of PIEZO1 in RBC volume homeostasis was confirmed by the observation that mice or zebrafish RBC knockdown for *piezo1* was hyperhydrated compared to WT RBC (Faucherre et al., 2014; Cahalan et al., 2015). Moreover, a patient with combined *PIEZO1* mutations correlated with decreased expression of the protein exhibited overhydrated RBC (Andolfo et al., 2015).

The present data combined with the previous publications reinforce the difficulty to link hemolytic anemia to KCNN4 gain-of-function mutations. The activity of the mutated channel appeared to be most of the time under strict control, kept silent in RBC with one notable exception, the V282M mutation that yields a constitutive K^+^ leaky channel that is clearly associated with RBC dehydration (Rivera et al., 2017). Since 2015, and taking into account the new mutations reported here, 11 different KCNN4 mutations have been identified in independent kindreds (P204R, A279T, S314P, V222L, V282/M or E, H340N, R352H, and delV369-L373) (Andolfo et al., 2015; Glogowska et al., 2015; Rapetti-Mauss et al., 2015; Fermo et al., 2017; Utsugisawa et al., 2017; Picard et al., 2019; Fermo et al., 2020; Andolfo et al., 2021; Mansour-Hendili et al., 2021). According to the different patients studied so far, using the RBC hydration status to diagnose a DHSt associated to KCNN4 mutations is perhaps misleading. The dehydration seems to be a signature of PIEZO1 gain-of-function mutations and exceptionally of KCNN4 mutation, when the mutation converts the channel into a constitutive K^+^ leak (the V282M mutation is the only one documented so far). Nonetheless, the presence of a gain-of-function mutated Gardos channel in RBC can dramatically impair RBC viability by a mechanism that remains to be identified. This fragility might involve other KCNN4 functions than permeability or require additional events. The susceptibility for a given KCNN4 gain-of-function mutation to lead to hemolysis appears more dramatic in early life, which is a classical feature of constitutional RBC membrane hemolytic anemia. More experimental data will be needed to better understand the physiological role of KCNN4 and how the mutations alter its function in RBC.

## Data Availability

The data presented in the study are deposited in the SRA repository, accession number PRJNA853732.

## References

[B1] Abraham M. J. Murtola T. Schulz R. Páll S. Jeremy C. Hess S. B. (2015). GROMACS: High Performance Molecular Simulations through Multi-Level Parallelism from Laptops to Supercomputers. SoftwareX 1-2, 19–25. 10.1016/j.softx.2015.06.001

[B2] Albuisson J. Murthy S. E. Bandell M. Coste B. Louis-Dit-Picard H. Mathur J. (2013). Dehydrated Hereditary Stomatocytosis Linked to Gain-Of-Function Mutations in Mechanically Activated PIEZO1 Ion Channels. Nat. Commun. 4, 1884. 10.1038/ncomms2899 23695678 PMC3674779

[B3] Amendola L. M. Jarvik G. P. Leo M. C. McLaughlin H. M. Akkari Y. Amaral M. D. (2016). Performance of ACMG-AMP Variant-Interpretation Guidelines Among Nine Laboratories in the Clinical Sequencing Exploratory Research Consortium. Am. J. Hum. Genet. 99, 247. 10.1016/j.ajhg.2016.06.001 27392081 PMC5005465

[B4] Andolfo I. Alper S. L. De Franceschi L. Auriemma C. Russo R. De Falco L. (2013). Multiple Clinical Forms of Dehydrated Hereditary Stomatocytosis Arise from Mutations in PIEZO1. Blood 121, 3925–3935. 10.1182/blood-2013-02-482489 23479567

[B5] Andolfo I. Martone S. Rosato B. E. Marra R. Gambale A. Forni G. L. (2021). Complex Modes of Inheritance in Hereditary Red Blood Cell Disorders: A Case Series Study of 155 Patients. Genes. (Basel) 12, 958. Epub 20210623. 10.3390/genes12070958 34201899 PMC8304671

[B6] Andolfo I. Russo R. Manna F. Shmukler B. E. Gambale A. Vitiello G. (2015). Novel Gardos Channel Mutations Linked to Dehydrated Hereditary Stomatocytosis (Xerocytosis). Am. J. Hematol. 90, 921–926. 10.1002/ajh.24117 26178367

[B7] Badens C. Guizouarn H. (2016). Advances in Understanding the Pathogenesis of the Red Cell Volume Disorders. Br. J. Haematol. 174, 674–685. Epub 2016/06/29. 10.1111/bjh.14197 27353637

[B8] Baux D. Van Goethem C. Ardouin O. Guignard T. Bergougnoux A. Koenig M. (2021). MobiDetails: Online DNA Variants Interpretation. Eur. J. Hum. Genet. 29, 356–360. Epub 20201107. 10.1038/s41431-020-00755-z 33161418 PMC7868358

[B9] Brugnara C. de Franceschi L. Alper S. L. (1993). Inhibition of Ca(2+)-dependent K+ Transport and Cell Dehydration in Sickle Erythrocytes by Clotrimazole and Other Imidazole Derivatives. J. Clin. Invest. 92, 520–526. 10.1172/jci116597 8326017 PMC293641

[B10] Cahalan S. M. Lukacs V. Ranade S. S. Chien S. Bandell M. Patapoutian A. (2015). Piezo1 Links Mechanical Forces to Red Blood Cell. Elife 4, e07370. 10.7554/eLife.07370 PMC4456639 26001274

[B11] Caulier A. Jankovsky N. Demont Y. Ouled-Haddou H. Demagny J. Guitton C. (2020). PIEZO1 Activation Delays Erythroid Differentiation of Normal and Hereditary Xerocytosis-Derived Human Progenitor Cells. Haematologica 105 (3), 610–622. 10.3324/haematol.2019.218503 31413092 PMC7049340

[B12] Coste B. Xiao B. Santos J. S. Syeda R. Grandl J. Spencer K. S. (2012). Piezo Proteins Are Pore-Forming Subunits of Mechanically Activated Channels. Nature 483, 176–181. 10.1038/nature10812 22343900 PMC3297710

[B13] Crooks G. E. Hon G. Chandonia J. M. Brenner S. E. (2004). WebLogo: a Sequence Logo Generator. Genome Res. 14, 1188–1190. 10.1101/gr.849004 15173120 PMC419797

[B14] Da Costa L. Suner L. Galimand J. Bonnel A. Pascreau T. Couque N. (2016). Diagnostic Tool for Red Blood Cell Membrane Disorders: Assessment of a New Generation Ektacytometer. Blood Cells Mol. Dis. 56, 9–22. Epub 20150916. 10.1016/j.bcmd.2015.09.001 26603718 PMC4811191

[B15] Danielczok J. G. Terriac E. Hertz L. Petkova-Kirova P. Lautenschläger F. Laschke M. W. (2017). Red Blood Cell Passage of Small Capillaries Is Associated with Transient Ca. Front. Physiol. 8, 979. Epub 2017/12/05. 10.3389/fphys.2017.00979 29259557 PMC5723316

[B16] De Franceschi L. Saadane N. Trudel M. Alper S. L. Brugnara C. Beuzard Y. (1994). Treatment with Oral Clotrimazole Blocks Ca(2+)-Activated K+ Transport and Reverses Erythrocyte Dehydration in Transgenic SAD Mice. A Model for Therapy of Sickle Cell Disease. J. Clin. Invest. 93, 1670–1676. 10.1172/jci117149 7512989 PMC294212

[B17] Dyrda A. Cytlak U. Ciuraszkiewicz A. Lipinska A. Cueff A. Bouyer G. (2010). Local Membrane Deformations Activate Ca^2+^-dependent K^+^ and Anionic Currents in Intact Human Red Blood Cells. PLoS One 5 (2), e9447. 10.1371/journal.pone.0009447 20195477 PMC2829085

[B18] Faucherre A. Kissa K. Nargeot J. Mangoni M. E. Jopling C. (2014). Piezo1 Plays a Role in Erythrocyte Volume Homeostasis. Haematologica 99, 70–75. 10.3324/haematol.2013.086090 PMC4007942 23872304

[B19] Fermo E. Bogdanova A. Petkova-Kirova P. Zaninoni A. Marcello A. Makhro A. (2015). Gardos Channel Mutation Is Associated with Hereditary Dehydrated Stomatocytosis: a Complex Channelopathy. Blood 126, 3333. 10.1182/blood.v126.23.3333.3333

[B20] Fermo E. Bogdanova A. Petkova-Kirova P. Zaninoni A. Marcello A. P. Makhro A. (2017). 'Gardos Channelopathy': a Variant of Hereditary Stomatocytosis with Complex Molecular Regulation. Sci. Rep. 11 (7), 1744. Epub 20170511. 10.1038/s41598-017-01591-w PMC5431847 28496185

[B21] Fermo E. Monedero-Alonso D. Petkova-Kirova P. Makhro A. Pérès L. Bouyer G. (2020). Gardos Channelopathy: Functional Analysis of a Novel KCNN4 Variant. Blood Adv. 12 22 (4), 6336–6341. 10.1182/bloodadvances.2020003285 PMC7756992 33351129

[B22] Flatt J. F. Bruce L. J. (2018). The Molecular Basis for Altered Cation Permeability in Hereditary Stomatocytic Human Red Blood Cells. Front. Physiol. 9, 367. Epub 2018/04/16. 10.3389/fphys.2018.00367 29713289 PMC5911802

[B23] Gallagher P. G. (2017). Disorders of Erythrocyte Hydration. Blood 12 (130), 2699–2708. Epub 2017/10/19. 10.1182/blood-2017-04-590810 PMC5746162 29051181

[B24] Gárdos G. Szász I. Sarkadi B. (1977). Effect of Intracellular Calcium on the Cation Transport Processes in Human Red Cells. Acta Biol. Med. Ger. 36, 823–829. 602587

[B25] Garneau L. Klein H. Banderali U. Longpré-Lauzon A. Parent L. Sauvé R. (2009). Hydrophobic Interactions as Key Determinants to the KCa3.1 Channel Closed Configuration. An Analysis of KCa3.1 Mutants Constitutively Active in Zero Ca^2+^ . J. Biol. Chem. 284, 389–403. 10.1074/jbc.m805700200 18996847

[B26] Girodon F. Garçon L. Bergoin E. Largier M. Delaunay J. Fénéant-Thibault M. (2008). Usefulness of the Eosin-5'-Maleimide Cytometric Method as a First-Line Screening Test for the Diagnosis of Hereditary Spherocytosis: Comparison with Ektacytometry and Protein Electrophoresis. Br. J. Haematol. 140, 468–470. Epub 20071219. 10.1111/j.1365-2141.2007.06944.x 18162119

[B27] Glogowska E. Lezon-Geyda K. Maksimova Y. Schulz V. P. Gallagher P. G. (2015). Mutations in the Gardos Channel (KCNN4) Are Associated with Hereditary Xerocytosis. Blood 126, 1281–1284. 10.1182/blood-2015-07-657957 26198474 PMC4566808

[B28] Glogowska E. Schneider E. R. Maksimova Y. Schulz V. P. Lezon-Geyda K. Wu J. (2017). Novel Mechanisms of PIEZO1 Dysfunction in Hereditary Xerocytosis. Blood 10130, 1845–1856. Epub 2017/07/17. 10.1182/blood-2017-05-786004 PMC5649553 28716860

[B29] Gnanasambandam R. Bae C. Gottlieb P. A. Sachs F. (2015). Ionic Selectivity and Permeation Properties of Human PIEZO1 Channels. PLoS One 10, e0125503. 10.1371/journal.pone.0125503 25955826 PMC4425559

[B30] Grygorczyk R. Mohandas N. (2015). More Than One Way to Shrink. Blood 126, 1263–1264. 10.1182/blood-2015-07-657916 26359428

[B31] Ji T. Corbalán-García S. Hubbard S. R. (2018). Crystal Structure of the C-Terminal Four-Helix Bundle of the Potassium Channel KCa3.1. PLoS One 13, e0199942. Epub 2018/06/28. 10.1371/journal.pone.0199942 29953543 PMC6023178

[B32] Kopanos C. Tsiolkas V. Kouris A. Chapple C. E. Albarca Aguilera M. Meyer R. (2019). VarSome: the Human Genomic Variant Search Engine. Bioinformatics 35, 1978–1980. 10.1093/bioinformatics/bty897 30376034 PMC6546127

[B33] Lee C. H. MacKinnon R. (2018). Activation Mechanism of a Human SK-Calmodulin Channel Complex Elucidated by Cryo-EM Structures. Science 360, 508–513. 10.1126/science.aas9466 29724949 PMC6241251

[B34] Maher A. D. Kuchel P. W. (2003). The Gárdos Channel: a Review of the Ca2+-Activated K+ Channel in Human Erythrocytes. Int. J. Biochem. Cell. Biol. 35, 1182–1197. 10.1016/s1357-2725(02)00310-2 12757756

[B35] Mansour-Hendili L. Egée S. Monedero-Alonso D. Bouyer G. Godeau B. Badaoui B. (2021). Multiple Thrombosis in a Patient with Gardos Channelopathy and a New KCNN4 Mutation. Am. J. Hematol. 96, E318–E321. Epub 20210602. 10.1002/ajh.26245 34004026

[B36] Mohandas N. Chasis J. A. (1993). Red Blood Cell Deformability, Membrane Material Properties and Shape: Regulation by Transmembrane, Skeletal and Cytosolic Proteins and Lipids. Semin. Hematol. 30, 171–192. 8211222

[B37] Mohandas N. Gallagher P. G. (2008). Red Cell Membrane: Past, Present, and Future. Blood. Nov. 112, 3939–3948. 10.1182/blood-2008-07-161166 PMC2582001 18988878

[B38] More T. A. Dongerdiye R. Devendra R. Warang P. P. Kedar P. S. (2020). Mechanosensitive Piezo1 Ion Channel Protein (PIEZO1 Gene): Update and Extended Mutation Analysis of Hereditary Xerocytosis in India. Ann. Hematol. 99, 715–727. Epub 20200228. 10.1007/s00277-020-03955-1 32112123

[B39] Olsson M. H. Søndergaard C. R. Rostkowski M. Jensen J. H. (2011). PROPKA3: Consistent Treatment of Internal and Surface Residues in Empirical pKa Predictions. J. Chem. Theory Comput. 7, 525–537. Epub 20110106. 10.1021/ct100578z 26596171

[B40] Picard V. Guitton C. Mansour-Hendili L. Jondeau B. Bendélac L. Denguir M. (2021). Rapid Gardos Hereditary Xerocytosis Diagnosis in 8 Families Using Reticulocyte Indices. Front. Physiol. 11, 602109. 10.3389/fphys.2020.602109 33519508 PMC7841495

[B41] Picard V. Guitton C. Thuret I. Rose C. Bendelac L. Ghazal K. (2019). Clinical and Biological Features in PIEZO1-Hereditary Xerocytosis and Gardos-Channelopathy: A Retrospective Series of 126 Patients. Haematologica 104 (8), 1554–1564. 10.3324/haematol.2018.205328 30655378 PMC6669138

[B42] Rapetti-Mauss R. Lacoste C. Picard V. Guitton C. Lombard E. Loosveld M. (2015). A Mutation in the Gardos Channel Is Associated with Hereditary Xerocytosis. Blood 126, 1273–1280. 10.1182/blood-2015-04-642496 26148990

[B43] Rapetti-Mauss R. Picard V. Guitton C. Ghazal K. Proulle V. Badens C. (2017). Red Blood Cell Gardos Channel (KCNN4): the Essential Determinant of Erythrocyte Dehydration in Hereditary Xerocytosis. Haematologica 102, e415–e418. Epub 2017/06/15. 10.3324/haematol.2017.171389 28619848 PMC5622875

[B44] Rapetti-Mauss R. Soriani O. Vinti H. Badens C. Guizouarn H. (2016). Senicapoc: a Potent Candidate for the Treatment of a Subset of Hereditary Xerocytosis Caused by Mutations in the Gardos Channel. Haematologica 101, e431–e435. Epub 2016/07/21. 10.3324/haematol.2016.149104 27443288 PMC5394861

[B45] Richards S. Aziz N. Bale S. Bick D. Das S. Gastier-Foster J. (2015). Standards and Guidelines for the Interpretation of Sequence Variants: a Joint Consensus Recommendation of the American College of Medical Genetics and Genomics and the Association for Molecular Pathology. Genet. Med. 17, 405–424. Epub 20150305. 10.1038/gim.2015.30 25741868 PMC4544753

[B46] Rivera A. Vandorpe D. Gallagher D. Fikry C. Kuypers F. Brugnara C. (2017). Erythrocytes from Hereditary Xerocytosis Patients Heterozygous for KCNN4 V282M Exhibit Increased Spontaneous Gardos Channel-like Activity Inhibited by Senicapoc. Am. J. Hematol. 92 (6), E108–E110. Epub 2017 Apr 29. 10.1002/ajh.24716 28295477

[B47] Rogers S. Lew V. L. (2021). Up-down Biphasic Volume Response of Human Red Blood Cells to PIEZO1 Activation during Capillary Transits. PLoS Comput. Biol. 17, e1008706. Epub 20210303. 10.1371/journal.pcbi.1008706 33657092 PMC7928492

[B48] Sievers F. Wilm A. Dineen D. Gibson T. J. Karplus K. Li W. (2011). Fast, Scalable Generation of High-Quality Protein Multiple Sequence Alignments Using Clustal Omega. Mol. Syst. Biol. 7, 539. Epub 20111011. 10.1038/msb.2011.75 21988835 PMC3261699

[B49] Søndergaard C. R. Olsson M. H. Rostkowski M. Jensen J. H. (2011). Improved Treatment of Ligands and Coupling Effects in Empirical Calculation and Rationalization of pKa Values. J. Chem. Theory Comput. 7, 2284–2295. Epub 20110609. 10.1021/ct200133y 26606496

[B50] Syeda R. Xu J. Dubin A. E. Coste B. Mathur J. Huynh T. (2015). Chemical Activation of the Mechanotransduction Channel Piezo1. Elife 4, e07369. 10.7554/eLife.07369 PMC4456433 26001275

[B51] Utsugisawa T. Iwasaki T. Aoki T. Okamoto Y. Kawakami T. Ogura H. (2017). The Flow Cytometric Osmotic Fragility Test Is an Effective Screening Method for Dehydrated Hereditary Stomatocytosis. Blood 130, 929. 10.1182/blood.v130.suppl_1.929.929

[B52] Waldstein S. Arnold-Croop S. Carrel L. Eyster M. E. (2021). Diagnosing Dehydrated Hereditary Stomatocytosis Due to a KCNN4 Gardos Channel Mutation: Understanding Challenges through Study of a Multi-Generational Family. eJHaem 2, 485–487. Epub 13 july 2021. 10.1002/jha2.267 35844691 PMC9175893

[B53] Webb B. Sali A. (2016). Comparative Protein Structure Modeling Using MODELLER. Curr. Protoc. Bioinforma. 54, 5.6.1–5.6.37. Epub 20160620. 10.1002/cpps.20 PMC5031415 27322406

[B54] Wu E. L. Cheng X. Jo S. Rui H. Song K. C. Dávila-Contreras E. M. (2014). CHARMM-GUI Membrane Builder toward Realistic Biological Membrane Simulations. J. Comput. Chem. 35, 1997–2004. Epub 20140807. 10.1002/jcc.23702 25130509 PMC4165794

[B55] Zarychanski R. Schulz V. P. Houston B. L. Maksimova Y. Houston D. S. Smith B. (2012). Mutations in the Mechanotransduction Protein PIEZO1 Are Associated with Hereditary Xerocytosis. Blood 120, 1908–1915. 10.1182/blood-2012-04-422253 22529292 PMC3448561

